# The core minimum dataset for measuring pain outcomes in pain services across Scotland. Developing and testing a brief multi-dimensional questionnaire

**DOI:** 10.1177/20494637221092907

**Published:** 2022-05-16

**Authors:** Magdalena S Laskawska, Harry L Hébert, Cara Richardson, Katherine Berlouis, Paul Cameron, Lesley A Colvin, Blair H Smith

**Affiliations:** 1 Chronic Pain Research Group, Division of Population Health and Genomics, Ninewells Hospital and Medical School, University of Dundee, Dundee, UK; 2 Fife Health & Social Care Partnership, Dunfermline, UK

**Keywords:** chronic pain, pain services, quality assessment, core outcome domains, questionnaire, validation

## Abstract

**Background:**

There is currently no agreed minimum dataset to inform specialist chronic pain service provision. We aimed to develop a Core Minimum Dataset (CMD) for pain services in Scotland and perform preliminary analysis to evaluate its psychometric properties in adults with chronic pain.

**Methods:**

The questionnaire was developed following a review of existing relevant data collection instruments and national consultation. The CMD questionnaire was completed alongside a routine pre-clinic questionnaire by patients attending two pain services over 3 months. Concurrent validity was tested by comparing scores between the CMD and pre-existing questionnaires. Reliability was assessed by test-retest and discriminative validity via receiver operating characteristic (ROC) curves.

**Results:**

The final CMD questionnaire consisted of five questions on four domains: pain severity (Chronic Pain Grade [CPG] Q1); pain interference (CPG Q5); emotional impact (Patient Health Questionnaire-2 [PHQ-2], two questions); and quality of life (Short Form Health Survey-36 [SF-36] Q1). 530 patients completed the questionnaire. Strong correlation was found with the Hospital Anxiety and Depression Scale (r_s_ = 0.753, *p* < 0.001). Moderate correlations were found with the Brief Pain Inventory for pain interference (r_s_ = 0.585, *p* < 0.001) and pain severity (r_s_ = 0.644, *p* < 0.001). Moderate to good reliability was demonstrated (Intra-class Correlation Coefficient = 0.572–0.845). All items indicated good discrimination for relevant health states.

**Conclusions:**

The findings represent initial steps towards developing an accurate questionnaire that is feasible for assessing chronic pain in adults attending specialist pain clinics and measuring service improvements in Scotland. Further validation testing, in clinical settings, is now required.

## Introduction

In Scotland, and across the world, chronic pain is the leading cause of disability^1,2^ linked negatively with depression, anxiety, sleep problems and quality of life.^3–6^ There are many challenges associated with measuring chronic pain and how its management is affected by use of clinical services. Across Scotland,^7^ and elsewhere,^8,9^ a wide variety of questionnaire instruments are used to assess the extent of pain and its impact. There is also a recognised variation in pain service delivery and data collection in Scotland.^7^ This variability creates difficulty in making accurate assessments of the severity and impact of chronic pain in people attending healthcare services, and therefore the resources required to address it. Furthermore, it makes it difficult to assess the effectiveness of any service provision and improvement initiatives, and to compare services in different areas and over time.

Attempts have been made to develop a standardised approach for measuring pain and the effectiveness of specialist pain clinics in the UK.^10–12^ These include an electronic-based system which was used to assess pain-related outcomes (e.g. pain and quality of life)^10^ and a more extensive set of patient reported outcome measure (PROMs) questionnaires.^12,13^ However, both studies suffered from low recruitment, low patient response, data entry difficulties and incompatibility with healthcare IT systems. The Faculty of Pain Medicine and British Pain Society commissioned an extensive review of commonly used instruments to be used in specialist pain services. This provides a brief practical guide for each questionnaire, but not a comprehensive analysis of which ones might be most suitable for use in routine clinical practice .^14^

Internationally, the Initiative on Methods, Measurement and Pain Assessment in Clinical Trials (IMMPACT) initiative was the first to provide recommendations on the core outcome domains for chronic pain for clinical research.^8^ However, the instruments recommended to assess these domains are unlikely to be practical in a clinical setting as they require completion, entry and analysis of many questionnaire items. The VAPAIN study aimed to provide recommendations that could be implemented in both research and clinical practice to assess the effectiveness of multidisciplinary therapy.^9^ However, although the VAPAIN panel reached a consensus on the core domain set for clinical trials, they have not yet been able to agree this for clinical practice (perhaps reflecting the complexity of this specialist area). Thus, further research is needed to identify reliable and valid instruments for measuring chronic pain in clinical practice, across domains, which are concise and easy to use in routine care.

Many instruments exist to assess pain, but each is generally specific to one domain (e.g. severity, mood and function) and/or includes too many questions for use in clinical practice. Therefore, a questionnaire is required that is brief, valid and reliable, covering the main recommended domains, to collect baseline and outcome data relating to people attending specialist pain services.

The main aims of the current study were:^1^ to develop a questionnaire capable of providing a Core Minimum Dataset (CMD) for use in routine clinical practice, building on previous work using a combination of a literature review, national consultation with key stakeholders and a review of current clinical approaches^15^; and^2^ to perform initial analysis to assess the psychometric properties of the single items included in the questionnaire.

## Methods

### Development of the questionnaire

It is intended that the questionnaire developed for this study will be used within specialist pain clinic settings and contribute to a CMD. Therefore, we adopted an approach that would produce a brief, simple and pragmatic questionnaire suitable for use within this setting in a reasonable timeframe. The instruments that were chosen for the CMD had to meet the following criteria:

1. Cover the relevant core domains outlined in the IMMPACT recommendations. These domains were^1^ pain,^2^ physical functioning,_^3^_ emotional functioning,^4^ participant ratings of improvement and satisfaction with treatment,^5^ symptoms and adverse events and^6^ participant disposition^8^.^[Bibr bibr8-20494637221092907]^ Domains 5 and 6 were considered to be mainly relevant to clinical trial studies, and therefore, these were not included as part of the CMD. In addition, we do not cover domain 4 directly but have included a quality-of-life item that can be compared to previous measurements to provide an overall assessment of improving or worsening pain.
2. Be part of a current, validated questionnaire.
3. Have gone through consultation with representatives of pain services from all 14 Health Boards in the National Health Service (NHS) of Scotland as well as third sector organisations and people with lived experience of pain.

The need to identify questions to cover the following areas was therefore agreed, based on the IMMPACT guidance^8^: demographics, pain duration, pain severity, emotional impact, functional impact, health-related quality of life, pain site and underlying diagnosis.

An initial non-systematic review of the literature and scoping exercise was conducted to investigate examples of good pain data collection practice from across the world and available instruments related to chronic pain outcomes. These included the electronic Persistent Pain Outcomes Collaboration (ePPOC) in Australia and New Zealand,^16^ the validation and application of a core set of patient-relevant outcome domains to assess the effectiveness of multimodal pain therapy (VAPAIN) study in Germany,^17^ the Quebec Pain Registry (QPR) in Canada^18^ and the Collaborative Health Outcomes Information Registry (CHOIR) in the USA.^19^ The ePPOC aims to improve the quality of care and outcomes for people with chronic pain in Australia and New Zealand by collecting a standard set of information through specialist pain services. CHOIR is an open source, open standard and free data collection software developed by Stanford in partnership with the National Institutes of Health to help clinicians in the USA collect qualitative information on pain patients. QPR is an administrative and research database which provides standardised data on chronic pain patients. VAPAIN aims to develop a core outcome domain set to assess the effectiveness of multimodal pain therapies. VAPAIN conducted systematic reviews of the literature prior to using Delphi consensus methods amongst pain experts and patients to determine a core set of domains. Instruments for assessing chronic pain outcomes were identified and grouped according to domain (e.g. pain severity and psychological functioning). A literature search was performed to determine the psychometric properties of each instrument, specifically reliability and validity. NHS librarians were also consulted regarding questionnaire licensing policy, to ensure availability for use within NHS settings. Further details on the development of the CMD questionnaire and associated considerations can be obtained from the NHS Research Scotland report.^15^

From the available instruments, validated questionnaires or individual items were selected for each of the identified areas, forming the first draft of the CMD questionnaire. The selected questionnaires and items were chosen, using a consensus-based approach (involving LAC, BHS, PC, CR and KB), to represent the best combination of evidence about psychometric properties while balancing the need for detail with burden of completion, administration and licensing considerations. The single-item global pain severity scale (Question 1 of the Chronic Pain Grade [CPG] questionnaire)^20^ was selected to assess pain severity; the single-item global CPG pain interference scale (Question five of the CPG questionnaire)^20^ was selected to assess pain interference; the Patient Health Questionnaire-2 (PHQ-2)^21^ was selected to assess emotional functioning; and the single-item health-related quality of life scale (Question 1 of the Short Form Health Survey [SF-36] questionnaire)^22^ was selected to assess health-related quality of life. Although the questionnaire instruments from which these items are drawn are known to be valid and reliable,^20,23–25^ the performance of these individual items and their performance when combined with each other was not known and could not be assumed.

The CMD questionnaire was sent out for consultations in two separate cycles to all NHS Pain Services in Scotland and to relevant third sector organisations, including people living with pain. It was accompanied by a questionnaire asking respondents to assess acceptability and feasibility of the dataset. Adaptations, based on feedback, were made after each cycle. There were 16 respondents in total to both consultations, from pain services in 9 of the 14 NHS Health Boards in Scotland; these 9 serve approximately 75% of the Scottish population. Their responses were collated and each was addressed by the project team, before subsequent drafts of the CMD questionnaire were developed. A copy of the consultation table which includes the comments and their responses can be found on the NHS Research Scotland website (http://www.nhsresearchscotland.org.uk/uploads/tinymce/Attachment1-Consultationtable.pdf). The final version of the CMD questionnaire contained six patient-completed clinical items drawn from four existing questionnaires, as well as five demographic items (age and gender, postcode, Community Health Index (CHI) number and current date) and diagnosis (to be completed by a clinician, based on the International Classification of Diseases, 11th edition [ICD-11]).^26^ The version of the CMD questionnaire, used in the subsequent validation exercise, is shown in Appendix S1.

### Sample and procedure

**Figure 1.**
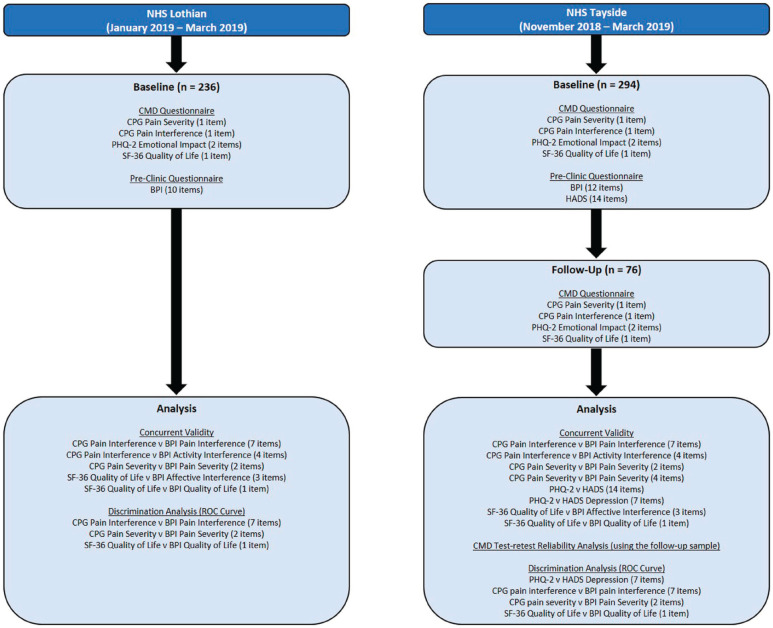
Flow diagram of patient recruitment, questionnaire administration and analysis. BPI, Brief Pain Inventory; CMD, Core Minimum Dataset; CPG, Chronic Pain Grade; HADS, Hospital Anxiety and Depression Scale; NHS, National Health Service; PHQ-2, Patient Health Questionnaire-2; ROC, receiver operating characteristic; SF-36, 36-item Short Form Survey.

A flow diagram of patient recruitment, questionnaire administration and analysis is provided in Figure 1. Patients with chronic pain, referred to two of the specialist NHS pain services in Scotland (Tayside and Lothian Health Boards), were asked to take part in the exercise by completing the CMD questionnaire along with the services’ routinely completed standard pre-clinic questionnaires. Patients were recruited sequentially to the study according to who attended the pain clinics and completed the CMD questionnaire. We wanted to test whether our questionnaire would be valid and reliable across different services, potentially using different methods for data collection. In NHS Tayside, all of the patients who received a referral to the specialist pain service were asked to complete a pre-clinic questionnaire pack, including the Brief Pain Inventory (BPI)^27^ and the Hospital Anxiety and Depression Scale (HADS)^28^ and to return the completed questionnaires within two weeks in order to be appointed for an outpatient assessment. The CMD questionnaire (Appendix S1) was also added to this pack along with a covering letter in which this was highlighted to the patients. In NHS Lothian the CMD and covering letter were included with the pre-clinic questionnaire that patients completed with assistance from a nurse, if required, at their first pain clinic appointment. In NHS Lothian, the standard pre-clinic questionnaire included the BPI (minus two items on pain severity: see The Standard Questionnaires For Comparison section), but did not include the HADS. Here, nurses provided patients with the CMD questionnaire and informed them that participation in this questionnaire was voluntary. Data collection was conducted between November 2018 and March 2019 in NHS Tayside (with a break for national holidays meaning that the duration of data collection was 3 months) and between January and March 2019 in NHS Lothian. A convenience sample of patients completed the CMD questionnaire again at the point of initial clinic attendance in NHS Tayside, prior to any treatment recommendations, so that response comparisons could be made with their pre-posted questionnaires. The CHI number was used to match the initial and the test-retest CMD questionnaires. The CHI number is a unique number that identifies each patient registered with the NHS in Scotland and it is attached to all clinical records. The Project Assistant, who held an honorary NHS contract, was responsible for entering, and anonymising, the data obtained from the patients from both test sites into an Excel spreadsheet. An anonymised dataset was sent to the University of Dundee, for analysis, using the secure and encrypted email service.

### Instruments

#### Core minimum dataset questionnaire (Appendix S1)

Reponses to the four patient-completed clinical items (from three existing questionnaires) were compared with relevant scales that were available from the standard validated questionnaires used routinely in each of the two Pain Services (Table 1).

**Table 1.** Comparison of items in the core minimum dataset questionnaire with validated instruments used in two specialist pain services.

Domain	CMD item	Validated questionnaires
Pain severity	CPG Q1: ‘In the past three months, on average, how intense was your pain rated on a 0–10 grade scale where 0 is “no pain” and 10 is “pain as bad as it could be”’ Potential response: 0–10	Brief pain Inventory (pain severity scale)
Pain interference	CPG Q5: ‘In the past six months, how much has this pain interfered with your daily activities rated on a 0–10 scale where 0 is “no interference” and 10 is “unable to carry on activities”’ Potential response: 0–10	Brief pain Inventory (pain interference and activity interference scale)
Emotional impact	PHQ-2: ‘Please circle the option that applies to you. Over the past 2 weeks, how often have you been bothered by any of the following problems? 1. Little interest or pleasure in doing things 2. Feeling down, depressed or hopeless’. Potential response: 0 - not at all, 1 - several days, 2 - more than half the days, 3 - nearly every day	Hospital anxiety and depression scale
Quality of life	SF-36 Q1: ‘Please circle the option that applies to you. In general, would you say that your health is’. Potential response: 1 - Excellent, 2 - Very good, 3 - Good, 4 - Fair, 5 - poor	Brief pain Inventory (affective interference scale and the single-item enjoyment of life scale)

CMD, core minimum dataset; CPG, chronic pain grade; PHQ-2, Patient Health Questionnaire-2; SF-36, short form health survey-36.

The single-item scales from the CMD included the following: CPG = Chronic Pain Grade Questionnaire (questions one and five)^20^ PHQ-2 = Patient Health Questionnaire-2^21^ and SF-36 = Short Form Health Survey.^22^ The single-item pain severity score can range from 0 to 10 (0 indicates ‘no pain’ and 10 indicates ‘pain as bad as it could be’). The single-item pain interference scale score can range from 0 to 10, (0 indicates *‘*no interference’ and 10 indicates ‘unable to carry on activities’). The PHQ-2 score can range from 0 to 6 (0 = ‘not at all’, 1 = ‘several days’, 2 = ‘more than half the days’, 3 = ‘nearly every day’). The single-item quality of life score ranges from 1 to 5 (1 indicates ‘excellent’ and five indicates ‘poor’). Table 1 provides the question wording for each of the items included in the CMD.

#### The standard questionnaires for comparison

##### The Hospital Anxiety and Depression Scale^28^

The Hospital Anxiety and Depression Scale (HADS) is a 14-item scale designed to assess depression (7 items) and anxiety (7 items). Each item of the HADS is rated on a 4-point scale ranging from 0 to 3. The maximum score for each scale is 21 and scores of 0–7 are considered as being normal, 8–10 suggests borderline abnormal depression or anxiety and 11–21 is indicative of severe depression or anxiety.^28^ The HADS is known to be valid and reliable in a general population^29^ and in a population of people with pain.^30^ The HADS is commercially licensed and a fee must be paid to use it.

##### The Brief Pain Inventory^27^

The The Brief Pain Inventory (BPI) is a self-administered instrument used to assess pain severity and pain interference. The short form of the BPI consists of nine questions: one on the presence of pain on the day of completion (Q1), one on the location of pain (using a body map; Q2), four on pain severity (worst pain, least pain, average pain and current pain; Q3–6), two on pain treatment and relief (Q7–8) and one on pain interference (Q9 split into seven items: general activity, mood, walking ability, normal work, relationship with other people, sleep and enjoyment of life). Only the four pain severity questions and pain interference question (with seven items) were used in this study. Each of these items are scored from 0 to 10, 10 indicating the most adverse state. Additionally, measures of activity interference, affective interference and quality of life were taken from combinations of the pain interference question (see following section on questionnaire scoring). The BPI pain severity and pain interference sub-scales were previously found to be valid and reliable in a population of people with chronic pain.^31^ The affective interference, activity interference and quality of life measures were selected pragmatically as the instruments that we thought were the closest match for the questions in the CMD, based on what was available as part of the pain services’ pre-existing questionnaires. An application for permission to use the BPI must be obtained from the copyright holder (Charles S. Cleeland) and a fee may be payable.

### Questionnaire scoring

We used a standardised method of scoring to calculate the HADS and the BPI scores.^27,28^ The NHS Lothian pain service used a modified BPI in their pre-clinic questionnaire, with only two out of four pain severity items in BPI (Q3 and Q5 of the short form BPI). The following items were included in NHS Lothian: ‘Please rate your pain by marking the box beside the number that best describes your pain at its worst in the last 24 h’ and ‘Please rate your pain by marking the box beside the number that best describes your pain on the average’. Therefore, the total BPI pain severity score was calculated by adding the scores from these two pain severity items. Additionally, the BPI pain interference score was calculated by the sum of the seven interference items (Q9: A-G), whilst the BPI pain activity interference was calculated by the sum of the general activity (Q9: A), walking ability (Q9: C), normal work (Q9: D) and sleep (Q9: F) items and the BPI affective interference was calculated by the sum of the mood (Q9: B), relationship with other people (Q9: E) and enjoyment of life (Q9: G) items. The enjoyment of life item (Q9: G) was taken as a proxy for quality of life.

The HADS overall emotional distress score was calculated from the sum of all 14 items in the screening tool and the HADS depression sub-scale score was calculated from the sum of the 7 depression items in the screening tool.

### Statistical analysis

A prospective power calculation was carried out in G*power 3.1.9.4 for Mac using exact correlation: bivariate normal model.^32^ This indicated that a minimum sample size of 84 (from each of the two services) would be required to run the correlation analyses (the power level [1–β] was equal to 80%, the alpha level [α] was set at 0.05 and the effect size [d] was equal to 0.3). A medium effect size was chosen for this sample size calculation to reflect the uncertainties around the tools being correlated, as well as the practical considerations around recruiting participants.^33^

Concurrent validity was tested by correlating the scores from items in the CMD questionnaire with relevant scores from the pre-existing validated questionnaires, focussing on those that were predicted to be assessing the same or similar domain, and that would therefore give the best assessment of the CMD item’s validity. Spearman’s rank analysis was therefore conducted between the following items:

1. The single-item global CPG pain interference scale and the BPI pain interference scale /BPI activity interference sub-scale
2. The single-item global CPG pain severity scale and the BPI pain severity scale. Two comparisons were conducted as a sensitivity analysis to account for the reduced number of items used in the BPI pain severity scale in NHS Lothian; one using both NHS Tayside and NHS Lothian (two-item BPI severity) and one using NHS Tayside only (4-item BPI severity).
3. The PHQ-2 and the HADS depression sub-scale/HADS overall emotional distress (NHS Tayside only).
4. The single-item SF-36 quality of life and BPI affective interference sub-scale/BPI single-item quality of life sub-scale.

A non-parametric correlation coefficient was applied because the assumption of normality was not satisfied for most of the variables. Spearman’s correlation coefficients (r) below 0.4 were categorised as ‘weak’; between 0.4 and 0.7 were categorised as ‘moderate’ and those over 0.7 were categorised as ‘strong’.^34,35^ The pairwise exclusion (available cases analysis) method was implemented in this study because it allowed us to use as many cases as possible when computing each statistic.

Test-retest reliability was evaluated in the NHS Tayside sample using the Intra-class Correlation Coefficient (ICC) (two-way mixed effects, absolute agreement and multiple measurements model)^36^ and limits of agreement (Bland–Altman method).^37^ Raw scores from the single-item pain severity scale and the single-item pain interference scale (both CPG) were transformed using log 10 with reflection to eliminate negative skewness. The ICC was performed on log-transformed scores for the single-item pain severity and pain interference scales and on raw scores for the two-item depression scale (PHQ-2) and single-item global quality of life scale (SF-36). ICC values below 0.5 were defined as ‘poor’, between 0.5 and 0.75 were defined as ‘moderate’, between 0.75 and 0.9 were defined as ‘good’ and those over 0.9 were defined as ‘excellent’.^38^ The Bland–Altman analysis was used to assess the agreement between the test and retest scores for the CMD.^37^ The level of agreement was evaluated by examining the mean differences between the two readings (1^st^ reading and 2^nd^ reading) and 95% limits of agreement were calculated as the mean difference ±1.96 x standard deviation. Since the same tools were being used to produce the repeated assessments, it was expected that the mean difference between the 1st and 2nd readings would be 0. This was assessed by conducting a one-sample T-test (two-tailed) with the significance threshold set at *p* <0.05. Rejection of the null hypothesis would indicate that the mean difference differed significantly from 0 and the tool was not reliable.

The discriminatory ability of the CMD was evaluated using Receiver Operating Characteristic (ROC) analysis. The area under the ROC curve (AUC) can range from 0 to 1 (with a value less than 0.5 indicating ‘worse than chance’ performance and a value of 1 representing a ‘perfect test’); hence, it is used to determine the diagnostic accuracy of the CMD.^39,40^ The interpretation of the accuracy of the CMD questionnaire was based on the recommendations proposed by Fischer et al. 2003.^41^ An AUC value of greater than 0.9 indicated ‘high’ accuracy; values between 0.7 and 0.9 indicated ‘moderate’ accuracy; and values between 0.5 and 0.7 represented ‘low’ accuracy.

The PHQ-2 scale was tested against the HADS (depression scale) using the ROC curve. The authors of the HADS questionnaire recommended that a score of ⩾11/21 should be used to identify people who suffer from severe depression.^28^ Therefore, a cut-off score of >10 was used to divide people into those categorised with severe depression and those who were not. Additionally, the single-item pain interference scale (CPG) was tested against the BPI pain interference scale, the single-item pain severity scale (CPG) was tested against the two-item BPI pain severity scale and the single-item quality of life scale (SF-36) was tested against the BPI quality of life scale, using the ROC curve. The BPI user guide does not specifically state what score should be used to determine severe pain intensity or severe pain interference. However, a number of studies have analysed appropriate cut-offs for categorising the intensity and interference of pain based on a 0–10 numeric rating scale and have advocated using ⩾4 or 5/10 to define moderate or severe pain.^42–45^ We therefore determined a cut-off score of >10/20 to categorise people into those with and without severe pain, a cut-off score of >35/70 to classify people into those with and without severe pain interference and a cut-off score of >5/10 to classify people into those with and without severe disability. The sensitivity, specificity and precision values for each of these single-item response scales were also calculated.

All of the data were analysed in SPSS (Version 22, IBM).

### Approvals

National Caldicott Guardian approval for the use of patient identifiable data for secondary purposes was obtained for the first part of this study [approval no: 1516–0581] which involved the development of the CMD as well as the second phase [approval no: 1718–0329] which involved testing the validity and reliability of this dataset. Due to the terms of this approval, we were unable to transfer potential personally identifying information, such as demographic data, from the clinics for data analysis. This was to protect patient confidentiality. Local NHS approvals were obtained for this work, which was classified as service improvement. Specific ethical approval was not required, as confirmed by NHS Tayside and NHS Lothian Research and Development Departments.

## Results

A total of 530 patients participated in this project, of whom 236 were in NHS Tayside and 294 were in NHS Lothian areas. Seventy-five patients completed the follow-up questionnaires at the point of clinic attendance in NHS Tayside. In terms of administrative burden, the time needed to input each CMD questionnaire into Excel was less than 1 min, which was shorter than the time needed to input the validated questionnaires (approximately 4 min for the BPI, and 10 min for the HADS). The frequency of missing data in the CMD questionnaire at baseline was 13.8% (*n* = 73) for the single-item pain severity scale (CPG), 13.6% (*n* = 72) for the two-item depression scale (PHQ-2; first question), 13.4% (*n* = 71) for the two-item depression scale (PHQ-2; second question), 12.6% (*n* = 67) for the single-item pain interference scale (CPG) and 12.6% (*n* = 67) for the single-item quality of life scale (SF-36). In the follow-up questionnaire, there were no missing data for the single-item pain severity scale (CPG), the two-item depression scale (PHQ-2; first question), the single-item pain interference scale (CPG) and the single-item quality of life scale (SF-36). The frequency of missing data was 5.3% (*n* = 4) for the two-item depression scale (PHQ-2; second question). The frequency of missing data for the reference items at baseline was 9.3% (*n* = 22) for the HADS overall emotional distress (14 items), 6.4% (*n* = 15) for the HADS depression sub-scale (7 items), 37.9% (*n* = 201) for the BPI pain severity scale (2 items), 41.7% (*n* = 221) for the BPI pain interference scale (7 items), 39.8% (*n* = 211) for the BPI activity interference sub-scale (4 items), 39.2% (*n* = 208) for the BPI affective interference sub-scale (3 items) and 37.4% (*n* = 198) for the BPI Quality of Life sub-scale (1 item).

### Concurrent validity of the single- and two-item scales from the CMD

Table 2 shows the correlations between the items from the CMD questionnaire and the standard questionnaires. The results from NHS Tayside alone showed that the PHQ-2 two-item scale (which assesses depression/emotional impact) correlated strongly with the HADS depression sub-scale and the HADS overall emotional distress. In addition, the single-item 3-month global CPG pain severity scale was moderately correlated with the BPI pain severity scale. The results from NHS Tayside and NHS Lothian combined showed that the single-item global CPG pain interference scale was moderately correlated with the BPI pain interference scale and the BPI activity interference sub-scale. The single-item global CPG pain severity scale correlated moderately with the BPI pain severity scale. The single-item SF-36 quality of life scale was moderately correlated with the BPI affective interference scale and the single-item quality of life scale.

**Table 2.** Spearman’s correlation coefficient scores to test the concurrent validity of the items from the core minimum dataset questionnaire.

Questionnaire	Two-item depression scale (PHQ-2)	Single-item pain severity (CPG)	Single-item pain interference (CPG)	Single-item quality of life (SF-36)
HADS overall emotional distress; *N*=210^a^	0.77			
HADS depression sub-scale; *N*=216^a^	0.75			
BPI pain severity scale (two-item); *N* = 320		0.64		
BPI pain severity (four-item); *N* = 221^a^		0.63		
BPI pain interference scale (total); *N* = 305			0.59	
BPI activity interference sub-scale; *N* = 315			0.60	
BPI affective interference sub-scale; *N* = 318				0.54
BPI quality of life single-item sub-scale; *N* = 329				0.50

*Note:* All correlations *p* < 0.001.

a NHS Tayside only.

BPI, Brief Pain Inventory; CPG, Chronic Pain Grade; HADS, Hospital Anxiety and Depression Scale; NHS, National Health Service; PHQ-2, Patient Health Questionnaire-2; SF-36, SF-36, Short Form Health Survey-36;

The number of participants ranged from 210 to 329 depending on tests. Most of the missing data were related to the BPI questionnaire in NHS Lothian, as described above.

### Test-retest reliability of the single- and two-item scales from the CMD

Table 3 shows the ICCs for the CMD questionnaire. The ICCs scores were rated ‘good’ for the two-item depression scale (PHQ-2; ICC = 0.83), single-item pain severity scale (CPG; ICC = 0.79) and the single-item global quality of life scale (SF-36; ICC = 0.85) and ‘moderate’ for the single-item pain interference scale (CPG; ICC = 0.57). Table 4 shows the summaries of the Bland-Altman statistics for the CMD and includes the mean difference between the two readings (PHQ-2 = 0.24; CPG pain severity = 0.16; CPG pain interference = 0.13; SF-36 = −0.05) and the limits of agreement (Table 4) for all the items. None of the items from the CMD had a mean difference value that was significantly different from zero (*p* <0.05), meaning that all the items were reliable.

**Table 3.** Intra-class Correlation Coefficients scores to assess the test-retest reliability of the items from the core minimum dataset questionnaire completed at two separate times.

CMD	N	ICC	95% Confidence interval	
			Lower	Upper
1. Two-item depression scale (PHQ-2)	71	0.83	0.73	0.89
2. Single-item pain severity scale (CPG)	74	0.79	0.66	0.87
3. Single-item pain interference scale (CPG)	75	0.57	0.32	0.73
4. Single-item global quality of life scale (SF-36)	75	0.85	0.75	0.90

CMD, core minimum dataset; ICC, intra-class correlation coefficient.

**Table 4.** Bland–Altman statistics to assess the agreement between test and retest scores for the CMD questionnaire, comparing responses at two separate times.

Instruments	Mean difference^a^	95% Limits of agreement		P-value^b^	N
		Lower	Upper		
1. Two-item depression scale (PHQ-2; range 0–6)	0.24	−2.69	3.17	0.18	71
2. Single-item pain severity scale (CPG; range 0–10)	0.16	−2.14	2.47	0.24	74
3. Single-item pain interference scale (CPG; range 0–10)	0.13	3.00	3.12	0.49	75
4. Single-item global quality of life scale (SF-36; range 1–5)	−0.05	−1.44	1.39	0.52	75

a Mean Difference between the first and the second reading (bias).

b Null hypothesis is that the mean difference between the first and second reading is zero.

CMD, core minimum dataset; CPG, chronic pain grade; PHQ-2, Patient Health Questionnaire-2; SF-36, short form health survey-36

### Sensitivity and specificity of a single-item pain severity scale (CPG), single-item pain interference scale (CPG) and two-item emotional impact scale (PHQ-2)

**Figure 2.**
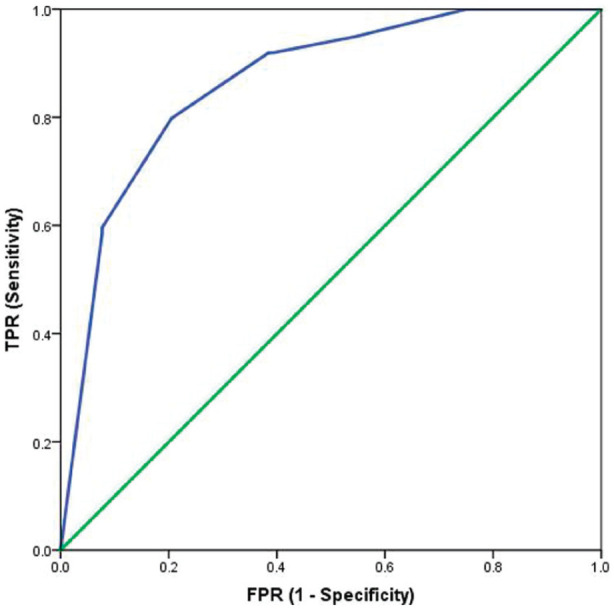
Receiver operating characteristic curve for the PHQ-2 as a screening tool: sensitivity and specificity of the two-item depression scale tested against the reference test (HADS-D >10/21). AUC = 0.87. *N* = 216.

**Figure 3.**
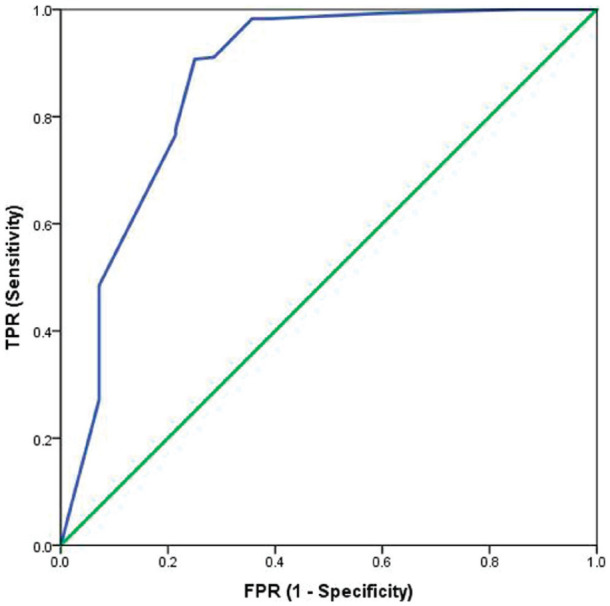
Receiver operating characteristic curve for the single-item global CPG pain severity scale as a screening tool: sensitivity and specificity of the single-item global CPG pain severity scale tested against the reference test (BPI pain severity scale >10/20). AUC = 0.87. *N* = 320.

**Figure 4.**
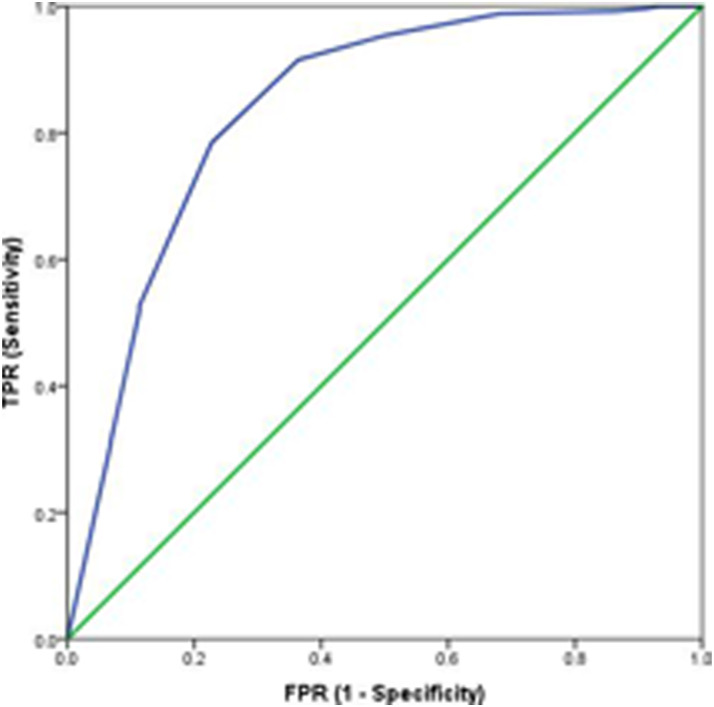
Receiver operating characteristic curve for the single-item global CPG pain interference scale as a screening tool: sensitivity and specificity of the single-item global CPG pain interference scale tested against the reference test (BPI pain interference scale >35/70). AUC = 0.84. *N* = 305.

**Figure 5.**
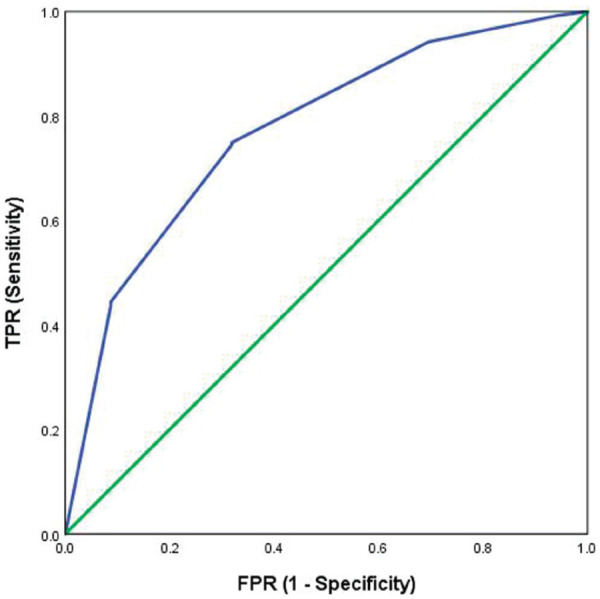
Receiver operating characteristic curve for the single-item SF-36 quality of life scale as a screening tool: sensitivity and specificity of the single-item SF-36 quality of life scale tested against the reference test (BPI quality of life sub-scale) >5/10). AUC = 0.77. *N* = 328.

Figures 2, 3, 4 and 5 illustrate the predictive validity of the two-item PHQ-2 scale, the single-item global CPG pain severity scale, the single-item global CPG pain interference scale and the single-item SF-36 scale. The area under the ROC curve was equal to 0.87 for the PHQ-2 scale (95% CI: 0.82 to 0.91), 0.84 for the single-item global CPG pain interference scale (95% CI: 0.77 to 0.91), 0.87 for the single-item global CPG pain severity scale (95% CI: 0.78 to 0.96) and 0.77 (95% CI: 0.71 to 0.83) for the single-item SF-36 scale. The optimal screening cut-off score was ⩾5 for the PHQ-2 scale (sensitivity = 80%, specificity = 80% and precision [positive predictive value] = 77%), the optimal cut-off point was ⩾8 for the single-item global CPG pain interference scale (sensitivity = 79%, specificity = 77% and precision = 95%), the optimal screening cut-off score was ⩾7 for the single-item global CPG pain severity scale (sensitivity = 91%, specificity = 75% and precision = 97%) and the optimal cut-off point was ⩾4 for the single-item SF-36 scale (sensitivity = 75%, specificity = 68% and precision = 90%).

## Discussion

### Summary

To the best of our knowledge, this is the first study to report on the development and initial testing of a single questionnaire for measuring the clinical characteristics of patients attending specialist pain services, in Scotland. Single-items scales from the CPG (Q1 and 5), PHQ-2 and SF-36 (Q1) were included in the questionnaire and had good psychometric properties (concurrent validity, reliability and discriminate ability). Further, more detailed analysis is required to validate the questionnaire in the clinical setting, using more comprehensive and validated tools as comparisons and assessing properties such as readability and time taken to complete the questionnaire.

### Interpretation and context

A Spearman’s correlation test revealed that the PHQ-2 correlated strongly with the Hospital Anxiety and Depression Scale. These results are in line with previous studies which showed that the PHQ-2 was positively associated with well validated assessments of depression and quality of life.^21,24,46^ Thus, it can be concluded that the PHQ-2 is a valid screening tool for depression in this group of patients.

Concurrent validity of the single-item pain interference scale was demonstrated by the moderate correlations with both the overall BPI pain interference scale, which is designed to assess both activity and affective interference of pain, as well as the BPI activity interference scale. Similarly, the single-item quality of life scale demonstrated moderate correlation with the BPI affective interference scale and BPI quality of life scale. Concurrent validity of the single-item pain severity scale was confirmed by the moderate correlation with the BPI pain severity sub-scale. These results support the use of our single-item global pain interference scale as an assessment tool for physical functioning. It should be noted that whilst the BPI was originally designed for use in people with cancer, it has also been validated in populations with non-malignant pain.^31,47^

It was found that the pain severity scale is a valid tool for assessing pain intensity. However, caution is advised when interpreting the results for the single-item CPG pain severity as this instrument relates to pain in the previous 3 months, whereas the available instrument in the pre-clinic questionnaires that was used for comparison (the BPI pain severity) related to pain in the previous 24 h. Further work should be conducted to test the single-item CPG pain severity against a similar tool assessing pain severity over 3 months. The receiver operating characteristic analyses confirmed the ability of scores relating to depression, pain severity, pain interference and health-related quality of life to detect clinically meaningful levels of these traits. The two-item depression scale demonstrated moderate accuracy (AUC = 0.87). The original validation study of PHQ-2 recommended a cut-off point of ⩾3 on the basis of a sensitivity of 0.83 and specificity of 0.90 for diagnosing major depressive disorder, with an AUC of 0.93.^21^ Similarly, the single-item pain severity, the single-item pain interference scale and the single-item health-related quality of life scale indicated moderate accuracy in our analysis (AUC = 0.87; AUC = 0.84; AUC = 0.77).

Finally, test-retest reliability was confirmed, noting that clinical states may themselves have altered during the interval between administrations of the questionnaire due to the dynamic nature and history of pain with variations in painful experiences. This may be relevant when interpreting the wide confidence intervals for some of the CMD items, particularly the single-item CPG pain interference. The analysis may therefore underestimate the test-retest reliability of the CMD items. This area should be analysed in more detail in future studies. The PHQ-2 scale demonstrated good reliability (ICC = 0.83) and this finding is consistent with previous studies in various populations.^48,49^ In addition, the ICCs for the single-item scales from the CMD questionnaire varied from 0.57 to 0.84, indicating moderate to good reliability.

The short time needed to enter the CMD into an electronic spreadsheet means that it imposes only a minimal administrative burden on pain service personnel. Unfortunately, previous studies do not report on the time burden of entering data from their questionnaires and so direct comparison is difficult. However, it is interesting to note that the Faculty of Pain Medicine and British Pain Society guidelines describe the HADS as ‘easy to score’.^14^ In this study we found that data from the HADS took approximately 10 min to enter into a spreadsheet. The guidelines also reported that it takes up to 10 min to complete the SF-36, though this is for the whole instrument, rather than a single item. We did not measure the time taken to calculate a score for each instrument in the study, nor was it possible to measure the time taken for each participant to complete the questionnaires. These are important considerations when designing a questionnaire for use in an everyday pain clinic setting and should be explored in future studies. However, as three of the four domains in the CMD are single items (pain severity, pain interference and quality of life), and a fourth has only 2 items (quality of life), little scoring will be necessary. This contrasts with other more complex and validated instruments, including those with which we compared the CMD (HADS and BPI). We hope that this simplicity will make it easier for the CMD to eventually be integrated into a computer-based data collection for specialist pain services.

The frequency of missing data in the CMD questionnaire at the baseline ranged from 12.6% to 13.8%, which although substantial, is comparable to that in other questionnaires.^11,13^ The CMD questionnaire is therefore a feasible instrument for collecting these pain-related data, but consideration needs to be given to maximising completion. Methods advocated in previous studies include focussing on resolving technical issues so that clinicians can access databases for data entry with minimal disruption and the supervision of patients completing forms. However, the later may introduce response bias as the most enthusiastic clinics may be less likely to have incomplete data.^11,13^ Further work should be conducted to explore the reasons for the missing data in the CMD questionnaire and potential solutions to ensure the risks of incomplete data are mitigated.

### Limitations

This study suffers from several limitations. As this study was conducted in specialist pain services and did not select participants based on any particular demographic or clinical characteristic, the results may not be generalisable to other settings such as primary care clinics. Moreover, only two specialist pain clinics participated in this validation exercise. Thus, it is unknown whether the results can be applied to specialist pain services elsewhere. However, these two pain clinics (NHS Lothian and NHS Tayside) currently use different methods for collecting their data and the CMD questionnaire proved to be valid and reliable across both services. Only one previous study has made an attempt to validate its approach to collecting pain service data, by comparing to written clinical notes.^11^ Future analysis should explore the differences between different demographic and clinical subgroups when they become available. The responsiveness to treatment of the CMD questionnaire was not evaluated in this study. Therefore, further longitudinal research is needed to investigate this.

Another limitation comes from the fact that the CMD is not yet embedded in routine electronic health records. Thus, our next objective is to develop a digital approach to data collection for the CMD. This in consequence will allow us to implement the CMD questionnaire across all specialist pain services in Scotland. Furthermore, there are many additional outcome domains that can be considered when assessing pain, such as those recommended by the VAPAIN team and other researchers.^50^

Although the single-item scales used in the questionnaire are part of larger validated instruments (CPG, PHQ-2 and SF-36), their use individually and combined with other items has not previously been validated; nor have the larger instruments been used in previous questionnaires and databases designed to assess pain services. In contrast, the instruments used to validate the CMD questionnaire (HADS and BPI) have been used in previous studies^10,13,14,16^, although we note that there is uncertainty around the use of HADS for separate assessments of depression and anxiety.^51^

However, the main objective of this study was to develop a validated national data collection tool which is brief, easy to administer and less burdensome to both patients and clinicians when compared with longer standardised questionnaires which are currently used in clinical practice, but often not entered into electronic records in a way that allows them to be used at the service level, rather than just the individual. Separately, we also developed an ‘optimum dataset’ which contains recommendations for additional clinical data collection, and the questionnaire instruments that could be used. This dataset can be accessed via the following link: http://www.nhsresearchscotland.org.uk/uploads/tinymce/NationalOutcomesSummaryReport-pain.pdf.

Finally, we could use only two (out of four) items of the BPI pain severity sub-scale to determine pain intensity, because these are all that were collected routinely by the service in NHS Lothian. Therefore, the validity of our reference test in this form cannot be assured. However, a sensitivity analysis was conducted to confirm validity. Furthermore, due to the lack of available quality of life instruments in both health boards, the BPI affective sub-scale was used to test the validity of the global quality of life scale. Therefore, future research should validate the global quality of life scale against standardised quality of life tools to confirm its validity. Similarly, the discriminative ability of the single-item global quality of life scale should also be evaluated.

### Conclusion

In conclusion, this study describes the development and initial testing of a CMD questionnaire for use in assessing chronic pain in adults attending specialist pain services in Scotland. It provides a foundation for more detailed analysis to validate the questionnaire. Preliminary findings suggest the items in the CMD are correlated with standard instruments, are reliable and can discriminate patients based on pain-related outcomes. In addition, the CMD is brief and less burdensome for clinicians entering data when compared with longer validated questionnaires currently in use. It is intended that the CMD questionnaire will eventually be embedded in routine clinical practice and enable monitoring and comparison of pain services. It is recommended that ongoing validation testing be conducted during this implementation. Future work will develop a digital approach to data collection for the CMD in collaboration with NHS Scotland and the Scottish Government, and this will allow ongoing testing in the clinical setting. The implementation of this standardised tool for measuring chronic pain will help us to reduce variation in service provision, provide us with a better understanding of the patients who currently use these types of specialist pain services and facilitate evaluation of outcomes and service improvement initiatives in Scotland.

## Supplemental Material

sj-pdf-1-bjp-10.1177_20494637221092907 – Supplemental material for The core minimum dataset for measuring pain outcomes in pain services across Scotland. Developing and testing a brief multi-dimensional questionnaire Click here for additional data file. Supplemental material, sj-pdf-1-bjp-10.1177_20494637221092907 for The core minimum dataset for measuring pain outcomes in pain services across Scotland. Developing and testing a brief multi-dimensional questionnaire by Magdalena S Laskawska, Harry L Hébert, Cara Richardson, Katherine Berlouis, Paul Cameron, Lesley A Colvin and Blair H Smith in British Journal of Pain
