# Supplementary material for: The core minimum dataset for measuring pain outcomes in pain services across Scotland. Developing and testing a brief multi-dimensional questionnaire
Source: Br J Pain. 2022 May 16;16(5):504–17. doi: 10.1177/20494637221092907 (PMC9644105; doi:10.1177/20494637221092907)
Supplement: sj-pdf-1-bjp-10.1177_20494637221092907 – Supplemental material for The core minimum dataset for measuring pain outcomes in pain services across Scotland. Developing and testing a brief multi-dimensional questionnaire [file sj-pdf-1-bjp-10.1177_20494637221092907.pdf]

The Core Minimum Dataset for measuring pain outcomes in services in Scotland. Developing and testing a brief multi-dimensional questionnaire.

Appendix S1 – The Core minimum dataset questionnaire

Demographics

1) Today's date

____

2) CHI Number

____

3) Please write your age

____

4) Gender

☐ Female

☐ Male

☐ Non-binary/ third gender

☐ Prefer to self-describe

____

☐ Prefer not to say

5) Postcode

____

6) Pain Duration

☐ Less than 3 months

☐ More than 3 but less than 6 months

☐ More than 6 but less than 12 months

☐ More than 1 but less than 3 years

☐ More than 3 but less than 6 years

☐ More than 6 but less than 10 years

☐ More than 10 years

7) Pain Severity

In the past three months, on average, how intense was your pain rated on a 0-10 grade scale where 0 is "no pain" and 10 is "pain as bad as it could be".

☐ 0 No Pain ☐ 1 ☐ 2 ☐ 3 ☐ 4 ☐ 5 ☐ 6 ☐ 7 ☐ 8 ☐ 9 ☐ 10 Pain as bad as it could be.

8) Emotional Impact

Please circle the option that applies to you.

Over the past 2 weeks, how often have you been bothered by any of the following problems?	Not At All	Several Days	More Than Half the Days	Nearly Every Day
1. Little interest or pleasure in doing things	0	1	2	3
2. Feeling down, depressed or hopeless	0	1	2	3

9) Functional Impact

In the past six months, how much has this pain interfered with your daily activities rated on a 0-10 scale where 0 is "no interference" and 10 is "unable to carry on activities"

☐ 0	☐ 1	☐ 2	☐ 3	☐ 4	☐ 5	☐ 6	☐ 7	☐ 8	☐ 9	☐ 10
No interference										Unable to carry on activities

10) Health-Related Quality of Life[1]

Please circle the number that applies to you.

In general, would you say that your health is:

Excellent	1
Very good	2
Good	3
Fair	4
Poor	5

[1]SF-36 is reproduced here (in part) with permission from the RAND Corporation. Copyright © the RAND Corporation. RAND's permission to reproduce the survey is not an endorsement of the products, services, or other uses in which the survey appears or is applied.

11) Pain Site (tick any/all that apply)

☐ Head

☐ Facial/Dental

☐ Cervical Spine

☐ Upper Limb: Shoulder/Arm/Wrist/Hand

☐ Chest

☐ Thoracic Spine

☐ Abdomen

☐ Lumbar/Sacral

☐ Pelvis

☐ Lower Limb: Hip/Buttock/Ankle/Foot

☐ Widespread

12) Underlying Diagnosis[2] (please refer to the ICD-11 classification section (below) for further details).

Please tick all of the options that apply.

☐ Chronic primary pain

☐ Chronic cancer pain

☐ Chronic postsurgical and posttraumatic pain

☐ Chronic neuropathic pain

☐ Chronic headache and orofacial pain

☐ Chronic visceral pain

☐ Chronic musculoskeletal pain

☐ Complex regional pain syndrome

---

[2] Proposed ICD-11 classification (Treede et al, 2015)

1. Chronic primary pain is pain in 1 or more anatomic regions that persists or recurs for longer than 3 months and is associated with significant emotional distress or significant functional disability (interference with activities of daily life and participation in social roles) and that cannot be better explained by another chronic pain condition.
2. Chronic cancer pain includes pain caused by the cancer itself (the primary tumour or metastases) and pain that is caused by the cancer treatment (surgical, chemotherapy, radiotherapy, and others).
3. Chronic postsurgical and posttraumatic pain is pain that develops after a surgical procedure or a tissue injury (involving any trauma, including burns) and persists at least 3 months after surgery or tissue trauma.
4. Chronic neuropathic pain is caused by a lesion or disease of the somatosensory nervous system.
5. Chronic headache and chronic orofacial pain is defined as headaches or orofacial pains that occur on at least 50% of the days during at least 3 months.
6. Chronic visceral pain is persistent or recurrent pain that originates from the internal organs of the head and neck region and the thoracic, abdominal, and pelvic cavities.
7. Chronic musculoskeletal pain is defined as persistent or recurrent pain that arises as part of a disease process directly affecting bone(s), joint(s), muscle(s), or related soft tissue(s).
8. Complex regional pain syndrome (CRPS) CRPS is preceded by a noxious event and is characterized by spontaneous pain or hyperalgesia/hyperaesthesia and other features, not limited to a single nerve territory and disproportionate to the inciting event.
